# The structural ensemble of a Holliday junction determined by X-ray scattering interference

**DOI:** 10.1093/nar/gkaa509

**Published:** 2020-06-29

**Authors:** Thomas Zettl, Xuesong Shi, Steve Bonilla, Steffen M Sedlak, Jan Lipfert, Daniel Herschlag

**Affiliations:** Department of Physics, Nanosystems Initiative Munich, and Center for Nanoscience, LMU Munich, 80799 Munich, Germany; Department of Biochemistry, Stanford University, Stanford, CA 94305, USA; Department of Biochemistry, Stanford University, Stanford, CA 94305, USA; Department of Biochemistry, Stanford University, Stanford, CA 94305, USA; Department of Chemical Engineering, Stanford University, Stanford, CA 94305, USA; Department of Physics, Nanosystems Initiative Munich, and Center for Nanoscience, LMU Munich, 80799 Munich, Germany; Department of Physics, Nanosystems Initiative Munich, and Center for Nanoscience, LMU Munich, 80799 Munich, Germany; Department of Biochemistry, Stanford University, Stanford, CA 94305, USA; Department of Chemical Engineering, Stanford University, Stanford, CA 94305, USA; Department of Chemistry, Stanford University, Stanford, CA 94305, USA; Stanford ChEM-H, Stanford University, Stanford, CA 94305, USA

## Abstract

The DNA four-way (Holliday) junction is the central intermediate of genetic recombination, yet key aspects of its conformational and thermodynamic properties remain unclear. While multiple experimental approaches have been used to characterize the canonical X-shape conformers under specific ionic conditions, the complete conformational ensemble of this motif, especially at low ionic conditions, remains largely undetermined. In line with previous studies, our single-molecule Förster resonance energy transfer (smFRET) measurements of junction dynamics revealed transitions between two states under high salt conditions, but smFRET could not determine whether there are fast and unresolvable transitions between distinct conformations or a broad ensemble of related states under low and intermediate salt conditions. We therefore used an emerging technique, X-ray scattering interferometry (XSI), to directly probe the conformational ensemble of the Holliday junction across a wide range of ionic conditions. Our results demonstrated that the four-way junction adopts an out-of-plane geometry under low ionic conditions and revealed a conformational state at intermediate ionic conditions previously undetected by other methods. Our results provide critical information to build toward a full description of the conformational landscape of the Holliday junction and underscore the utility of XSI for probing conformational ensembles under a wide range of solution conditions.

## INTRODUCTION

Holliday junctions are fundamental nucleic acid structural motifs that play central roles in genetic recombination and other cellular processes (1–4) and have been developed as a widely used tool in DNA nanotechnology (5–7). Holliday junctions, and nucleic acid junctions in general, often rely on conformational changes to carry out their biological functions, either alone or as building blocks of larger nucleic acid and nucleic acid-protein complexes (4,8–13). Many proteins, such as junction-resolving enzymes, recognize and distort the structure of junctions, for example by stabilizing unstacked conformations, breaking central base pairs or changing the inter-duplex angle between the arms of stacked conformations (4,8,10). These enzymes are highly specific for the structure of the junction (13), and defining the underlying conformational landscapes or ensembles of Holliday junctions and other DNA/RNA junctions is required to fully understand the action of proteins that modify nucleic acid conformational landscapes.

**Figure 1.**
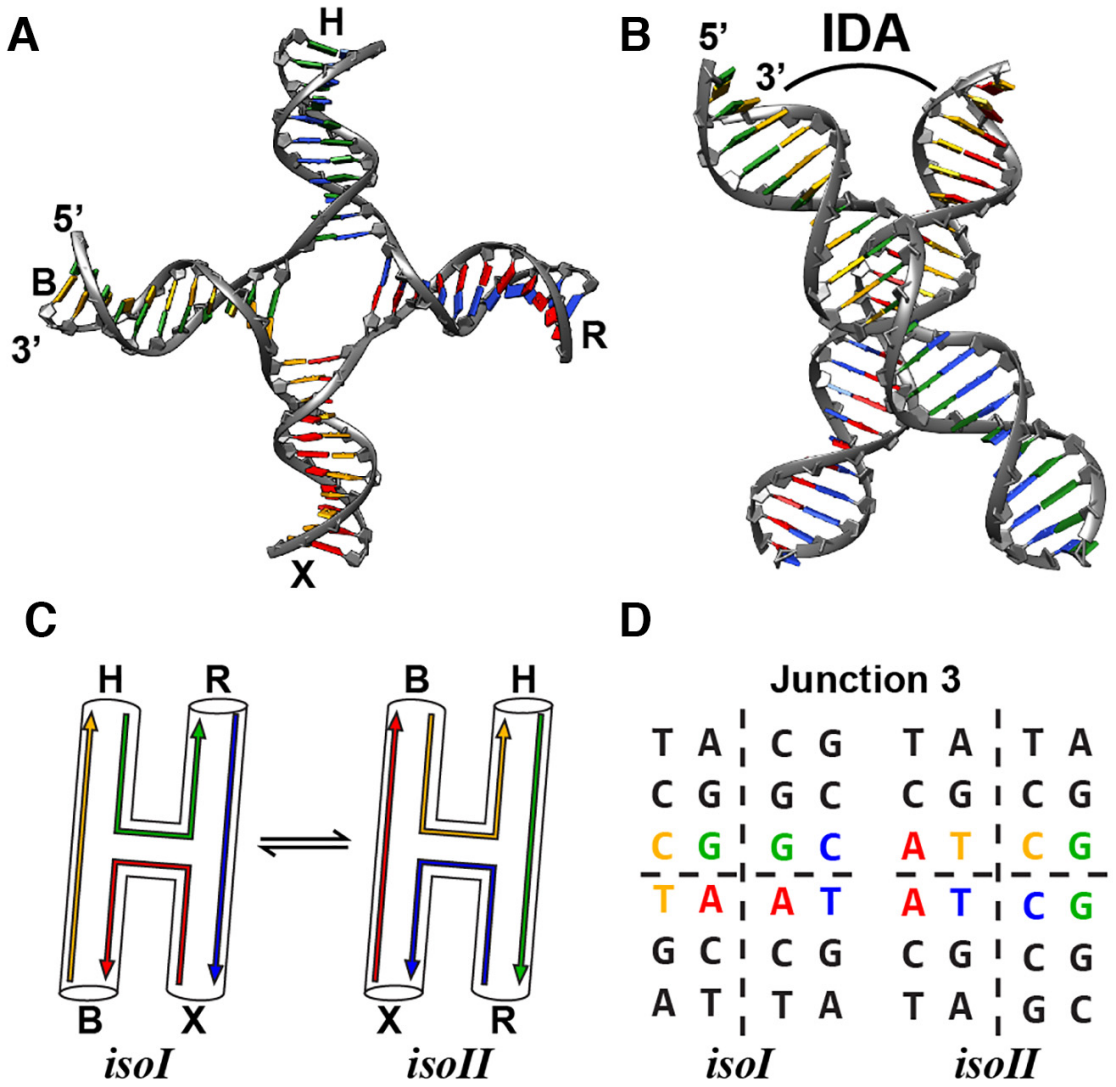
Structure of the Holliday junction. (**A**) Schematic of a four-way junction in an extended open conformation with the helical arms X, B, H and R annotated. The structure was rendered from the crystal structure obtained in the presence of a Cre recombinase (46) (PDB code: 2CRX). (**B**) Schematic of the stacked X-structure observed in the presence of counter-ions. Two right-handed helices are folded by coaxial stacking with an inter-duplex angle (IDA) on the order of ∼60° between arms (4,18,54). (**C**) Schematic of the two anti-parallel conformations isoI and isoII of the X-structure with different orientation of the helical arms X, B, H and R. (**D**) The central sequence of the junction motif 3 used in our study in both stacked states. The different colors visualize the individual pathways of the oligonucleotide strands in the two states.

Experimentally, it is known that an open state is predominant at low salt concentrations for a canonical Holliday junction, while the junctions adopt stacked conformations at higher ionic strength (9,11,14–17) (Figure 1A, B). These stacked conformations, which are induced through an interplay of stacking interactions, electrostatic repulsion between the negatively charged backbones, and electrostatic screening by salt ions, lead to the formation of two quasi-continuous helices with pairwise stacking of the helical arms (Figure 1B–D). There are two stacked conformations that differ in the choice of stacking partners within the central motif of the junction (11,14,18) (Figure 1C, D). Moreover, the choice of stacked conformation seems to play a key role in genetic recombination, as there is evidence that the adopted structure can influence the binding orientation of junction-resolving enzymes and therefore determine cleavage positions (10,13,14,19,20). While Holliday junctions have been extensively studied, important questions about their conformations and dynamics remain. Single-molecule Förster resonance energy transfer (smFRET) experiments of the Holliday junction have identified multiple states under solution conditions at high Mg^2+^ concentrations. Under high salt conditions, the conformational dynamics are slow enough to be resolved by conventional smFRET (11,16,17). However, conventional smFRET is limited to providing transition rates between resolved averaged states and it is difficult to inform on whether each state is dominated by a single rigid conformation or whether a broader conformational ensemble is present, as the steady-state FRET values are consistent with either case (21,22). To probe the ensemble of conformational states at intermediate and low salt concentration, we applied an emerging synchrotron based structural technique, X-ray scattering interferometry (XSI) (23–32). We used XSI with a model Holliday junction sequence under different salt conditions to address the following questions: (i) how does the distribution of Holliday junction states change with salt, (ii) what is the average solution structure and (iii) how broad is the ensemble of the major Holliday junction conformational states? We used these experimental data to generate and test physical models of Holliday junctions to demonstrate the change in the Holliday junction conformational landscape under different ionic environments.

We chose to examine the well-studied Holliday junction type 3 (14). Using the type 3 junction as a model system enabled us to compare our XSI data to prior published results obtained by various techniques including comparative gel electrophoresis (14,18), atomic force microscopy imaging (33), smFRET (11,16,17), small-angle X-ray scattering (34), and *in silico* studies using molecular dynamics simulations (35). XSI uniquely adds to this information by providing whole-ensemble distance distributions with Ångström resolution (23,24,26–32). It has been shown that under low ionic strength conditions (e.g. 30 mM Tris–HCl buffer only) the electrostatic repulsion between the DNA phosphate groups forces the Holliday junction motif such as junction type 3 to adopt in an open, unstacked state (4,14,18). Our XSI measurements confirm the presence of an open state and suggest that it is non-planar, likely with some degree of pyramidal character.

Under high ionic strength conditions (10 mM Mg^2+^) Holliday junctions tend to adopt stable, stacked conformations referred to in the literature as *isoI* and *isoII* (9,11,14,16,18) (Figure 1B, C). At intermediate ionic conditions (∼150 mM Na^+^ or ∼150 μM Mg^2+^), the electrostatic repulsion of the DNA backbone is sufficiently screened to allow the junction to adopt stacked conformations, which, however, remain dynamic (16,18) (Figure 1B, C). Our XSI data agree with the proposed X-shape model with the two stacked states at high and intermediate salt and provide additional structural and ensemble information about this Holliday junction, including evidence for a novel conformational state at intermediate salt conditions.

## MATERIALS AND METHODS

### Preparation of Gold-DNA conjugates

Gold nanocrystals with particle radius of 7 Å were synthesized and purified as described previously (23,24,32,36). Unmodified single-stranded DNA (ssDNA) was purchased from Integrated DNA Technology (IDT) and C3-thiol-modified oligonucleotides were synthesized at the Protein and Nucleic Acids Facility at Stanford University on an automated ABI 394 DNA synthesizer. All DNA sequences used in this study are reported in Supplementary Tables S1 and S2. Furthermore, ssDNA oligonucleotides were purified by ion exchange high-pressure liquid chromatography (HPLC) using a Dionex DNAPac 200 column and a salt gradient from low (10 mM NaCl) to high salt (1.5 M NaCl) supplemented with 20 mM sodium borate, pH 7.8. Gold-ssDNA conjugates were formed by mixing oligonucleotides with gold nanocrystals in a 1:5 ratio (ssDNA:gold nanocrystals) at room temperature in 100 mM Tris–HCl, pH 9.0 for two hours. Immediately after the gold attachment reaction conjugates were purified by HPLC using the Dionex anion exchange column and an elution gradient from 10 mM to 1.5 M NaCl supplemented with 20 mM ammonium acetate, pH 5.6 and desalted using Amicon centrifugal filters with 3 kDa cutoff (3500 × g with a swinging bucket rotor for 35 min; three repeats) at 4°C, to remove free gold, unlabeled DNA and gold particles with multiple DNA oligos attached.

DNA dimers (e.g. R and X form RX) were hybridized for 30 min at room temperature follow by HPLC purification using the same protocol as for the ssDNA gold nanocrystal conjugates. The full Holliday junction with two gold labels, one gold label and the unlabeled version were formed by mixing two equimolar complementary dimers (e.g. RX mixed with BH) in 10 mM MgCl_2_, 10 mM Tris–HCl, 1 mM EDTA, pH 8.0 and annealed by heating the solution to 65°C for 2 min followed by 39°C for another 10 min in a thermocycler (BioRad). Lastly, the fully assembled structures were purified similar to DNA dimers, desalted using 10K Dalton Amicon filters (3500 × g with a swinging bucket rotor for 15 min; three repeats) at 4°C and stored at −20°C until SAXS experiments. After each desalting step, concentrations were determined using a NanoDrop ND-1000 (NanoDrop Technologies) measuring the UV absorbance at 260 and 360 nm. Elution profiles and absorbance ratios were different for junctions without, with single and with double labels. We did not observe peaks that correspond to unlabeled oligos, individual single labeled oligos or dimers during the final purification.

### SAXS measurements and data processing

Small-angle X-ray scattering experiments were carried out at beamline 4-2 of the Stanford Synchrotron Radiation Ligthsource and BM29 at the European Synchrotron Research Facility in Grenoble. At beamline 4-2, data were collected using a detector distance of 1.1 m at 11 keV whereas at BM29 the detector distance was set to 2.867 m with an X-ray energy of 15 keV. At both beamlines, measurements used 30 μM final sample concentration. Recordings were performed at 15°C at beamline 4-2 and 5°C at beamline BM29. A buffer containing 30 mM Tris–HCl, pH 7.4 and 10 mM ascorbic acid was used to prevent radiation damage. This base buffer was supplemented with 10 mM MgCl_2_, 150 μM MgCl_2_, 1 M NaCl or 150 mM NaCl respectively for high or intermediate salt conditions or not supplemented with additional ions for the low salt measurements. The buffer exchange of the samples was performed using 10 kDa cutoff 0.5 ml Amicon filter units (14 000 × g for 25 min, repeated three times). Each sample was recorded in 10 × 3 s exposures and checked for ration damage. One full set of data for further analysis (Supplementary Figure S1) contains Holliday junction scattering profiles of a double labeled sample (AB), two single labeled samples (A label and B label), the unlabeled macromolecule (U), the gold nanocrystals (Au) and finally the buffer (Buf). The profiles are weighted, summed (AB and U) and subtracted (A and B) to calculate the gold–gold scattering interference pattern *I*_Au–Au_ (32). The resulting interference patterns were fitted using a maximum entropy algorithm to obtain the final distance distributions as described previously (23,24,36).

### Preparation of Dye-labeled DNA for single-molecule FRET

Single-stranded DNA–dye conjugates were prepared as described previously (37). Briefly, ssDNA with 3′-amino-modifier, 3′-biotin modified, and unmodified oligonucleotides were ordered from IDT. The sequences and modifications for all oligonucleotides are presented in Supplementary Tables S1 and S2. For dye labeling, the residual primary amines were removed using ethanol precipitation. After precipitation, the DNA was resuspended in a 100 mM phosphate buffer, pH 8.7. Cy3B and Cy5 were chosen as the FRET pair. Both NHS ester fluorophores were separately suspended in 3.5 μl DMSO and immediately afterwards 1 μl of dissolved dye was added to the corresponding aqueous oligonucleotide solution (strands R and X). The mixtures were incubated at 37°C for 1 h and both excess dye and unreacted ssDNA were removed using polyacrylamide gel-electrophoresis. Purified ssDNA-dye conjugates were extracted from the gel using squeeze and freeze. Finally, sample concentration was checked using a NanoDrop device. Furthermore, 3′-biotin modified oligonucleotides and unmodified ssDNA were purified using a Dionex DNAPac 200 column with a salt gradient ranging from low salt (10 mM NaCl and 20 mM sodium borate, pH 7.8) up to high salt (1.5 M NaCl and 20 mM sodium borate, pH 7.8). To assemble the full Holliday junction construct, all four complementary DNA strands (B, H, R and X) were mixed in an equimolar ratio and additionally a solution containing 10 mM Tris–HCl, 1 mM EDTA, 10 mM MgCl_2_, pH 8.0 was added. After mixing, the solution was heated to 65°C for 2 min followed by 39°C for 10 min. Finally, the construct was purified using ion exchange chromatography (Dionex DNAPac 200 column) and the concentration was determined using UV-absorbance at 260 nm.

### Single-molecule FRET measurements and data processing

Single-molecule FRET measurements were performed using a custom-built optical microscope setup and a widely-used preparation protocol (38). Flow channels for experiments were prepared as follows: Firstly, channels were filled with buffer containing 10 mM or 150 μM MgCl_2_, respectively, in 30 mM Tris–HCl at pH 7.4. Secondly, 12 μl of a 1 mg/ml Biotin-bovine serum albumin (BSA) solution was added and incubated for 4 min. Thirdly, the cell was washed with a buffer containing the specified salt condition. Moreover, the coverslip was coated with streptavidin such that a final density of ∼1 molecule/5 μm^2^ was achieved. The streptavidin mixture was incubated for 4 min. After incubation, the channel was washed with buffer containing the specified salt concentration again. Next, 2 pM of biotinylated Holliday junction sample was added and immobilized during the incubation for 4 min. Afterwards, unbound molecules were washed off with 20 volumes of buffer containing the specified salt condition. Finally, the channel was flushed with 2 volumes imaging buffer. Imaging buffers included the specified concentration of salt and in addition a standard oxygen scavenging system to slow down photobleaching including 2 mM protocatechuic acid (PCA), 0.001 units/μl protocatechuate-3,4-dioxygenase (PCD) and 1 mM Trolox (6-hydroxy-2,5,7,8-tetramethylchroman-2-carboxylic acid). Data were recorded at 306 frames per second and the measured time traces were analyzed using the SMART software package (39) (Supplementary Figure S2).

## RESULTS

To probe the solution conformations of a type 3 model Holliday junction, we modified the 3′ termini of identical oligonucleotide sequences with fluorescence dyes or gold nanoparticles, respectively, for smFRET or XSI measurements. In each case, the DNA junction was assembled from four synthetic oligonucleotides 22 bases in lengths (see Materials and Methods).

### Single-molecule FRET measurements of Holiday junction dynamics

**Figure 2.**
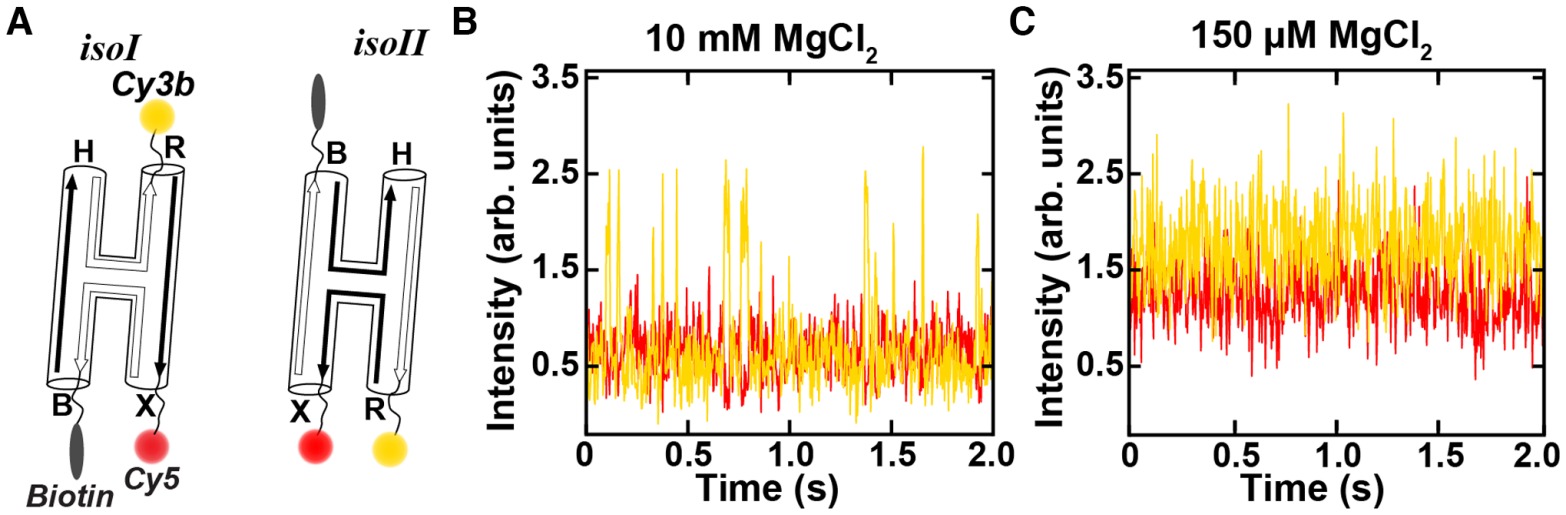
Single-molecule FRET constructs and time traces. (**A**) Schematic of the stacked states *isoI* and *isoII* of the Holliday junction including the incorporated dyes at the 3′ termini of the R (Cy3b) and X (Cy5) helices and a biotin label on helix B. (**B**) Typical smFRET trace recorded using a salt concentration of 10 mM MgCl_2_ and 30 mM Tris–HCl, pH 7.4. Transitions between the two states are visible as correlated changes in the donor and acceptor intensities. (**C**) Typical smFRET trace in a buffer containing 150 μM MgCl_2_ and 30 mM Tris. No clear transitions are visible for the low salt condition. Traces in panels (B) and (C) were recorded with a camera integration time of 3 ms.

We measured conformational changes of individual molecules in real-time to study the relative populations and the transition rates between the two stacked states of the model Holliday junction *isoI* and *isoII* (4,11,14,16) (Figure 2A). The oligonucleotides self-assemble from the four synthetic 22mer oligonucleotides into the desired four-way junction motif with four helical arms (X, B, H and R, Figure 1C) that cannot undergo branch migration (11). Two arms were conjugated with dyes at the 3′ termini to form a FRET pair, Cy3b as donor (arm R) and Cy5 as acceptor (arm X), and one arm was modified with biotin (arm B) for surface immobilization (Figure 2A). The junction has a large inter-dye distance in the first stacked conformation *isoI* which is expected to yield a low FRET value, whereas it has a small inter-dye distance in the second stacked conformation *isoII*, corresponding to an expected high FRET value (Figure 2A). The single molecule traces fluctuated between high and low FRET values of 0.58 and 0.14 respectively, consistent with the Holliday junction switching between *isoI* and *isoII*. At 10 mM MgCl_2_, the single molecules mostly populated the high FRET state (FRET = 0.58; Supplementary Figure S2), only interrupted by short intervals in the low FRET state (FRET = 0.14; Supplementary Figure S2). This observation is consistent with a strong bias towards the *isoII* state (Figure 2B). The rate constants for the transition between the conformations *k*_I__→__II_ and *k*_II__→__I_ were averaged over hundreds of transitions from 77 individual single-molecule traces and calculated to be 45.6 (±2.0) s^−1^ and 7.9 (±0.4) s^−1^, respectively, corresponding to populations of 15% *isoI* and 85% *isoII* (Supplementary Figure S2). Our results agree with previous smFRET studies reporting a population ratio of 1:4 (25% *isoI*: 75% *isoII*) at high divalent salt (11). The preference for stacked state *isoII* can be accounted for by a difference in free energy contribution from stacking of the central base pairs (*isoI*: TA/CG and CG/TA *vs*. *isoII*: AT/AT and GC/GC). Moreover, the transition rates measured here at 10 mM Mg^2+^ are faster than those reported at 50 mM Mg^2+^ (*k*_I__→__II_ = 12 s^−1^ and *k*_II__→__I_ = 3.5 s^−1^), in line with the proposed inverse dependence of transition rate on magnesium concentration (11,16).

We also recorded smFRET traces at lower Mg^2+^ concentration, using a buffer containing 150 μM Mg^2+^. Under these solution conditions, the FRET efficiency remained at a constant value and there were no clear transitions in the donor and acceptor signals (Figure 2C). This observation is consistent with the previous suggestion that the kinetic barrier between the two stacked states is lowered with decreasing salt concentration such that the transitions occur on a much faster timescale (<3 ms) to give an averaged FRET value (11,16). Consistent with this model, the average FRET value observed at low salt is intermediate between the two FRET states observed at 10 mM Mg^2+^ (Supplementary Figure S3). Nevertheless, the limited time resolution of our smFRET experiments prevents the observation of additional states of the Holliday junction under these conditions and does not distinguish whether the stacked states are the same as those that are present at high magnesium (*isoI* and *isoII*) or whether different or additional conformations are populated (11,16). We therefore turned to X-ray scattering interferometry (23–32).

### X-ray scattering interferometry

X-ray scattering interferometry (XSI) provides high-resolution information about ensemble distance distributions and can resolve models with a single conformation versus two (or more) rapidly interconverting complexes, while also providing structural information about each state (23,26–29,31). We therefore used XSI to study the Holliday junction at low salt concentrations, in the regime where transitions between putative states were not revealed by our smFRET measurements. XSI measures the interference scattering pattern between two site-specifically attached electron-rich markers together with scattering terms from the molecule and the cross-scattering terms between the labels and the macromolecule. The interference between the two labels is extracted from measurements of the doubly-labeled, singly-labeled, and unlabeled samples and transformed into an absolute distance distribution using a weighted linear combination of basis scattering functions (23,24,26,28,29,31,32).

**Figure 3.**
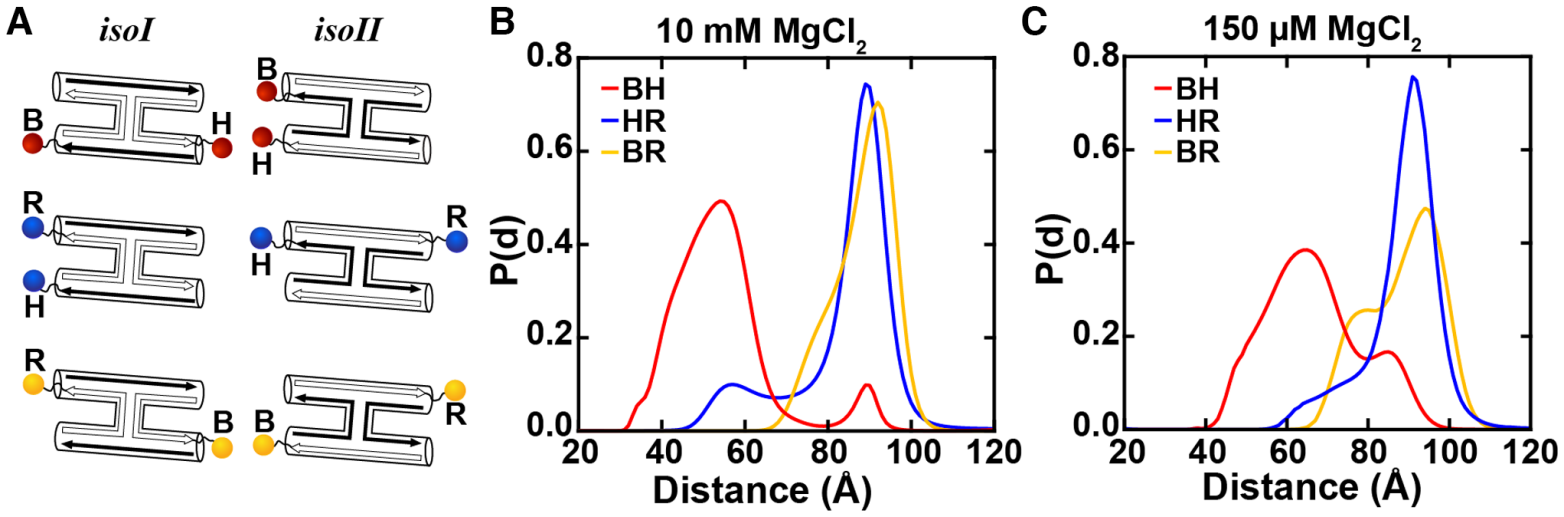
XSI Holliday junction constructs and measured distance distributions at high and intermediate divalent salt. (**A**) Schematic of the stacked states *isoI* and *isoII* of the Holliday junction and of the different label pairs (BH, HR, and BR). Distance distributions for the label pairs BH (red), HR (blue) and BR (yellow) recorded at (**B**) high (10 mM MgCl_2_ and 30 mM Tris, pH 7.4) and (**C**) intermediate divalent salt concentration (150 μM MgCl_2_ and 30 mM Tris, pH 7.4). The observation of multiple peaks is indicative of the presence of different states at each salt condition (see Supplementary Figures S4–S7, S8, S9). Distributions in (B) and (C) are normalized.

**Figure 4.**
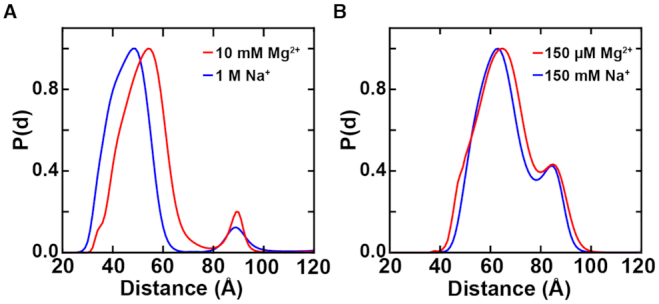
XSI distributions at high and intermediate monovalent salt. (**A**) Distance distributions for the label pair BH (see Figure 3A) recorded at high divalent salt (red, 10 mM MgCl_2_ and 30 mM Tris–HCl, pH 7.4) and high monovalent salt (blue, 1 M NaCl and 30 mM Tris–HCl, pH 7.4). (**B**) Distance distributions for the label pair BH recorded at intermediate divalent salt concentration (red, 150 μM MgCl_2_ and 30 mM Tris–HCl, pH 7.4) and intermediate monovalent salt (blue, 150 mM NaCl and 30 mM Tris–HCl, pH 7.4).

We assembled the DNA junction for XSI measurements from four synthetic oligonucleotides, with identical lengths and sequences as used in the smFRET experiments (see Materials and Methods). To provide the characteristic signal in XSI, we attached pairs of gold nanocrystal to generate four doubly labeled constructs (HR, BR, BH and RX, Figures 3 and 4) following published approaches (23,24,26,28,29,31,32) (see Materials and Methods). We also assembled, purified, and recorded data for the corresponding single-labeled and unlabeled constructs that are required to extract the gold-gold interference pattern.

Using XSI, we obtained gold–gold distance probability distributions for the Holliday junction across different salt conditions and using several pairs of labels (Figure 3A). Varying the attachment positions facilitated determination of the orientations adopted by the junction arms relative to one another and allowed us to distinguish between conformations of the different states. We observed that the distance distributions are dependent on the ionic condition.

### XSI measurements under high Mg^2+^ conditions reveal stable *isoI* and *isoII* conformations

Under conditions with high charge screening (10 mM MgCl_2_), we observed a major and a minor peak for each label pair. While the two peaks were clearly separated for the BH (Figure 3B, red line and Supplementary Figures S4, S5) and HR (Figure 3B, blue line Supplementary Figures S4, S6) label pairs, the two peaks overlapped significantly for BR (Figure 3B, yellow line, Supplementary Figures S4, S7). This behavior is qualitatively consistent with the expected differences in Au-Au distances between *isoI* and *isoII* (Figure 3A). Specifically, for BH the gold particles are expected to be far apart in *isoI* but close together in *IsoII* while the opposite is true for HR (Figure 3A). In contrast, for BR the difference in Au-Au distance between *isoI* and *isoII* is expected to be small due to the symmetry in Au label positions between these two conformations (Figure 3A).

The major population for the BH label pair centered around 54 Å and the minor second population, around 89 Å (Figure 3B, red line, Supplementary Figures S4, S5 and Table S3); conversely, for the HR labeled version the major peak was at 89 Å and the minor peak was at 57 Å (Figure 3B, blue line and Supplementary Figures S4, S6). These findings are in line with the proposed X-shape (Supplementary Figure S8), switching between two similar stacked conformers, and the reported bias for *isoII* from FRET studies (11,16). We estimated the average population of *isoI* (16%) and *isoII* (84%) by comparing the area under the XSI distance distributions (Supplementary Figure S4 and Table S4). The population ratio from the XSI measurements is in excellent agreement with the populations observed in our (15%:85%) smFRET measurements and in reasonable agreement with previous (25%:75%) smFRET studies (11), suggesting that the different labels in FRET vs. XSI and other experimental differences do not strongly affect the observed ensemble. The 89 Å distance for the BH and HR gold pairs is similar to that expected for a continuous helix of this length with gold labels at the opposite ends, supporting the presence of stacked configuration in both *isoI* and *isoII* (Figure 3B, red and blue lines, Supplementary Table S3; see also below).

In contrast to the constant distances for the *isoI* and *isoII* stacked helices of 89 Å, XSI revealed a small but significant difference between the short distance distribution of these label pairs for *isoI* and *isoII* (Figure 3B, BH and HR), suggesting that the junctions adopt slightly different configurations depending on the stacking partners. This difference could arise from different inter-duplex angles or from different rotations around the helical axes; such differences in base stacking angle would lead to a different rotation of the duplexed junction arm and thus to a change in radial displacement between the two gold probes. The end-to-end distance of 89 Å for opposite labels is shorter than the displacement of ∼93 Å predicted for a continuous B-form DNA helix (4) with helical rise of 3.3 Å (24,30,36) (to model Au linkers we used the known gold label offset parameters for dsDNA (23,24) and their salt dependence (29)). The smaller observed end-to-end distance compared to a continuous B-form helix suggests that in the Holliday junction the helix is overwound or bent to some degree. Bending—out of plane—may be more likely, due to electrostatic repulsion between the phosphates located along the backbone of neighboring helices.

### XSI at intermediate Mg^2+^ suggest loss of stacking interactions in *isoI*

We used the same gold nanocrystal pairs in 150 μM MgCl_2_ and 30 mM Tris–HCl, pH 7.4, to directly test how this Holliday junction motif responds to intermediate divalent salt (Figure 3C). As in high divalent salt, the junction preferred the *isoII* conformer (Figure 3C). The observed shift of the major peak from 89 to 91 Å (Figure 3C, blue line) for label pair HR (*isoII*) at 150 μM MgCl_2_ as compared to 10 mM MgCl_2_ (Figure 3B, blue line and Supplementary Figures S6, S9) agrees well with the previously reported small salt-dependent alteration (∼2 Å axial offset) in the gold nanocrystal position (29). There is a larger shift in the lower-distance major peak (*isoII*, label pair BH), from 54 to 61 Å (Figure 3C, red line, Supplementary Figures S5, S9 and Table S3). This increase is consistent with an increase in inter-duplex angle for a stacked *isoII* conformer due to lower electrostatic screening at the intermediate salt conditions.

The finding of a stacked conformer for the major population (corresponding to *isoII*) at 150 μM Mg^2+^ agrees with prior gel electrophoresis (18) and bulk FRET studies (16). However, the gold-gold distance peaks for BH and BR corresponding to conformer *isoI* (minor population) differ notably from distances observed at higher Mg^2+^ (Supplementary Figures S5, S6 and Table S3). Moreover, as the two BR label pair peaks should match for a highly symmetric structure, the large difference in these peaks for *isoI*, relative to *isoII* (Figure 3C, yellow line) and in contrast to the small difference of BR between *isoI* and *isoII* in higher salt (Figure 3B, yellow line and Supplementary Figure S7), suggests that the *isoI* structure is not symmetric in intermediate salt (Supplementary Table S3). The displacement to longer distances for the BH *isoI* peak suggests that the *isoI* junction is no longer stacked for one or both sets of helices as an increase in the Au-Au distance of only ∼2 Å for label pair BH is expected due to the Au linker salt dependency. The observed end-to-end distance of 84 Å for opposite labels of BH is significantly shorter than the displacement of ∼95 Å predicted for a continuous B-form DNA and increased Au linker offset parameters. According to the nearest-neighbor free energy parameters obtained from SantaLucia (40), the central base pairs contribute less stacking free energy to *isoI* than *isoII*. This lower gain in free energy from the central base pairs in *isoI* is presumably insufficient to overcome the electrostatic repulsion under this salt concentration for adoption of the stacked conformation.

### Holliday junction conformations in the presence of monovalent salt

To test whether the Holliday junction responds specifically to Mg^2+^ we obtained XSI data for high (1 M NaCl and 30 mM Tris–HCl, pH 7.4; corresponding roughly to the electrostatic screening potential of 10 mM MgCl_2_ (41–43)) and intermediate (150 mM NaCl and 30 mM Tris–HCl, pH 7.4) monovalent salt concentrations for the label pair BH, similar to experiments with MgCl_2_ (Figure 4). The results obtained at high sodium showed behavior similar, but not identical to that observed in the presence of high magnesium. We therefore conclude, in agreement with FRET studies (16), that the junction adopts the same two-state behavior at high monovalent ionic strength as it does with high divalent salt, forming the two well-stacked *isoI* and *isoII* states (Figure 4A, Supplementary Figure S10).

As expected based on polyelectrolyte behavior (41,43), much higher concentrations of monovalent than divalent cations were required to observe similar behavior (16,18). Moreover, data recorded at 150 mM NaCl indicate only one fully stacked state (Figure 4B, Supplementary Figure S10), as the peak located at ∼88 Å at high salt, corresponding to *isoI*, is shifted to a lower distance (84 Å), similar to the results in 150 μM MgCl_2_. As proposed for intermediate Mg^2+^ ion conditions, the lower gain in stacking free energy for *isoI* could account for an absence or modification of the *isoI* form.

### XSI measurements at low ionic strength suggest an unstacked and non-planar geometry

**Figure 5.**
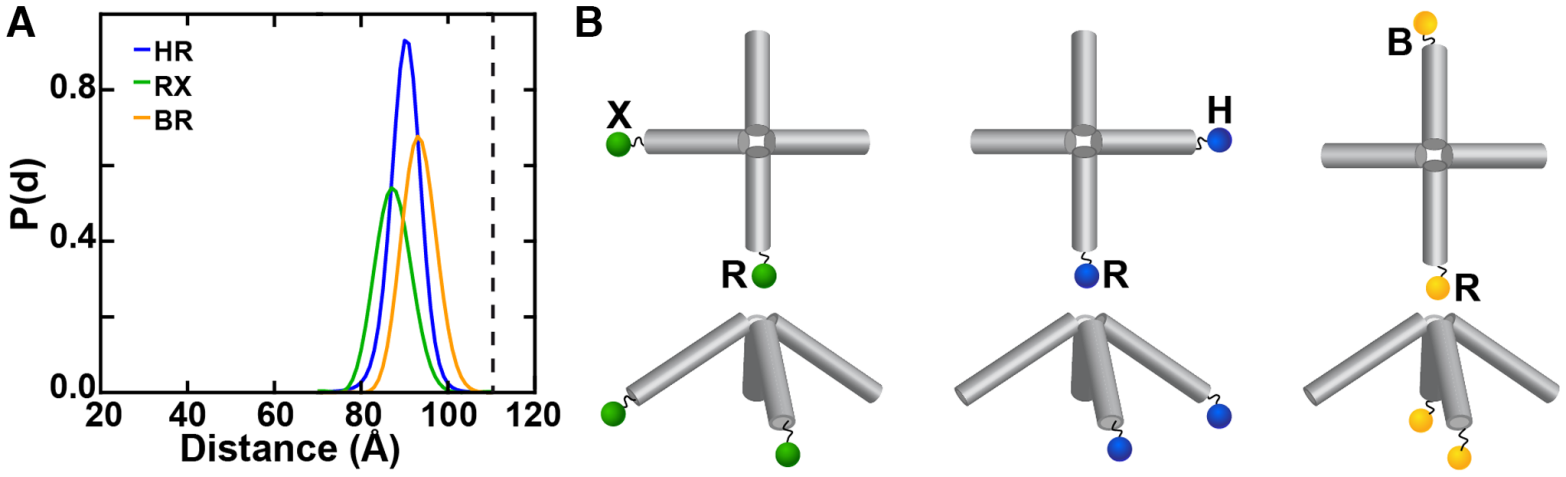
XSI distance distributions and schematic structures at low salt. (**A**) Distance distributions for the label pairs HR, RX, and BR recorded at low salt (30 mM Tris–HCl, pH 7.4, only). Distance distributions were normalized to a total probability of 1. The dashed line indicates the expected distance for BR of 111 Å for a square planar structure (compare to the yellow distribution). (**B**) Schematic for the label pairs from A) from top view (upper row) and side view (lower row).

To analyze the salt dependency further, we probed gold-gold distances under low salt conditions (30 mM Tris–HCl, pH 7.4). The label pairs HR and RX are orthogonal, such that if the molecules were folded into the stacked X-structure (Figure 1B), then one pair would show a high distance separation, while the other pair would exhibit a short distance. However, the gold-gold distances for each pair of labels are high, >80 Å (Figure 5). Moreover, only single peaks are observed for individual gold pairs HR, RX and BR, suggesting that there is no equilibrium formed between two stacked structures as occurs in high and intermediate ionic strength (Figure 5 and Supplementary Figures S6, S7). These results are consistent with prior FRET, gel-mobility, and FRET combined with hydrodynamic profiling data under low salt conditions and presumably reflect a conformational state in which electrostatic repulsion dominates over stacking (4,18,44,45). For the open state, a 2D planar geometry with 4-fold symmetry was suggested from AFM imaging (33) and SAXS (34), but others have suggested a 3D pyramidal conformation (4). As XSI provides precise information about distance distributions in solution, our data can be used to evaluate these models. The measured distance for BR of 93 Å (Figure 5 and Supplementary Figure S7) is less than the estimated ∼111 Å distance for a planar conformation. This value was obtained using a gap of 16.3 Å for the center, as predicted from a protein-bound planar crystal structure (46) (Figure 1A), a helical rise of 3.3 Å, and gold nanocrystal axial and rotational offset parameters from Mathew-Fenn *et al.* (24). The measured distance of 93 Å would only match a planar conformation if the arms formed a continuous helix with a 0 Å gap. However, the absence of a gap would correspond to a stacked structure. But, the 90 Å (HR) and 87 Å (RX) distances observed for the orthogonal label pairs HR and RX (Figure 5A) are inconsistent with a stacked structure, as the observed stacked structures give different distances along versus across the stacked helices (Figure 3), with a ∼35–37 Å difference between the two symmetric orthogonal label pairs (HR & BH) observed at high salt (Figure 3A, B and Supplementary Table S3). Thus, our experimentally determined distances suggest that the structure at low salt is open and non-planar.

In summary, our data recorded at low salt provide evidence against a planar 4-fold symmetric structure and are instead consistent with a pyramidal-like arrangement of the extended arms, possibly with some bending or deformations consistent with the slight differences between the RX and HR distances. We note that an overall similar structure has been observed in the presence of the junction-resolving enzyme GEN1 (47). Our data could also be consistent with a (slightly deformed) tetrahedral arrangement as proposed by simulations (48), which is, however, disfavored by gel mobility data (14).

More precise geometrical pictures of the conformational ensemble as well as information on how the ensemble changes in the low salt regime could be obtained from XSI experiments with additional Au-Au labels or by using single gold crystals as fiducial markers (49).

## DISCUSSION

We have used smFRET and XSI to investigate the solution ensemble of a Holliday junction motif. Consistent with prior FRET and gel-mobility studies, our findings indicated that at high salt concentrations the four-way junction preferentially adopts two distinct X-structures with coaxial pairwise stacking of helical arms, with a preference for the *isoII* conformation by ∼1 kcal/mol. Our results further demonstrate that the preferred conformation and thus the conformational free energy is highly salt-dependent. Also consistent with prior findings, our results suggest that solution conditions and the identities of the central base pairs can help sculpt the free-energy landscape of the Holliday junction.

Beyond that, our XSI data provide new conformational information about Holliday junctions. We found that the stacked structure for *isoI* differs from the stacked structure for *isoII* at high ionic strength. Conformational distributions at high and intermediate salt provided evidence for a novel state at intermediate salt. Furthermore, our results at low salt provided new insights into the geometry of the open structure, suggesting an open and non-planar conformation for the four-way junction with an asymmetric arrangement of the arms.

Our results underscore the ability of XSI to reveal precise ensemble properties across a wide range of ionic strength, even for rapidly interchanging conformations (28,29,32,36). XSI, therefore, will provide a valuable reference for emerging FRET approaches that provide calibrated distances (50) and ≤ms time resolution (45,51–53).

XSI lacks the atomic resolution attainable by some structural techniques, but provides accurate solution and, importantly, ensemble information. XSI is readily applicable to four-way junctions with proteins bound and we anticipate it to be a powerful tool to probe how protein binding-partners remodel the conformational landscape of DNA junctions. Beyond four-way DNA junctions, XSI is applicable to RNA junctions (29) as well as different junction topologies, e.g. three-way junctions (12) and has the potential to refine the conformational rules for these critical building blocks of higher order nucleic acid structures.

## Supplementary Material

gkaa509_Supplemental_File
