# Supplementary material for: The structural ensemble of a Holliday junction determined by X-ray scattering interference
Source: Nucleic Acids Res. 2020 Jun 29;48(14):8090–8. doi: 10.1093/nar/gkaa509 (PMC7641307; doi:10.1093/nar/gkaa509)
Supplement: gkaa509_Supplemental_File [file gkaa509_supplemental_file.pdf]

Supplementary Information for "The Structural Ensemble of a Holliday Junction Determined by X-Ray Scattering Interference"

Thomas Zettl[1,2], *Xuesong Shi*[2], *Steve Bonilla*[2,3], *Steffen M. Sedlak*[1], *Jan Lipfert*[1,*] *and Daniel Herschlag*[2,3,4,5,*]

[1]Department of Physics, Nanosystems Initiative Munich, and Center for Nanoscience, LMU Munich, Munich, Germany.
[2]Department of Biochemistry, Stanford University, Stanford, California 94305, USA.
[3]Department of Chemical Engineering, Stanford University, Stanford, California 94305, USA.
[4]Department of Chemistry, Stanford University, Stanford, California 94305, USA.
[5] Stanford ChEM-H, Stanford University, Stanford, California 94305, USA.

[*] To whom correspondence should be addressed. Tel: +1 650 723-9442; Fax: +1 650 723-6783 Email: herschla@stanford.edu

Correspondence may also be addressed to Jan Lipfert: Tel: +49 89 2180 2005; Fax: +49 89 2180 2050 Email: Jan.Lipfert@lmu.de

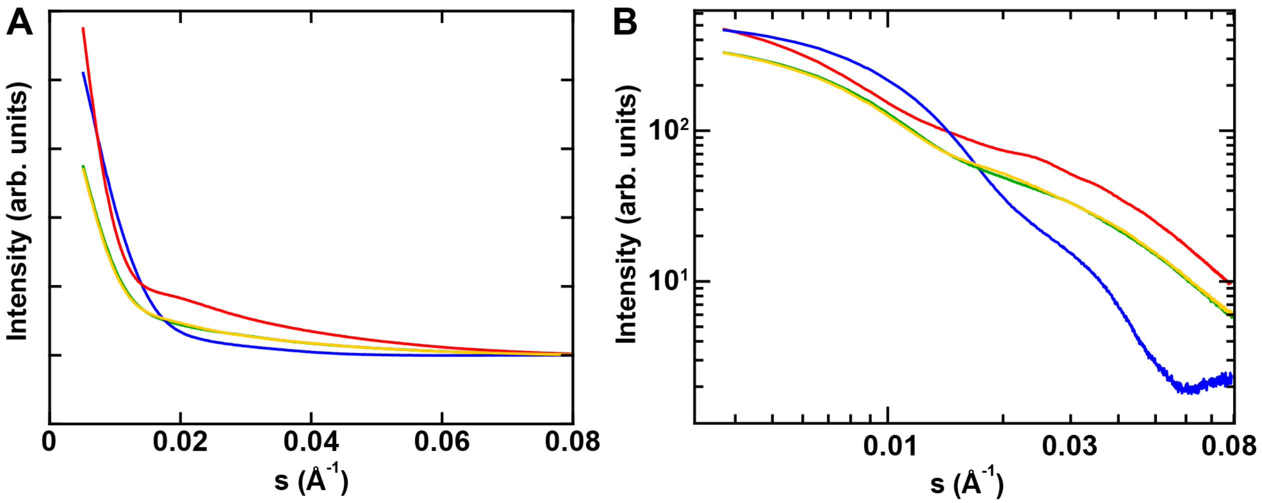

Supplementary Figure S1. Representative SAXS data sets used for obtaining XSI profiles. Data are shown in linear-linear representation (A) and as a log-log plot (B). The set contains SAXS profiles for constructs without (blue) and with single (green and yellow) or double (red) gold nanocrystal labels. The set of four profiles was used to calculate the XSI profile to extract the distances distribution as described in the **Materials and Methods** section.

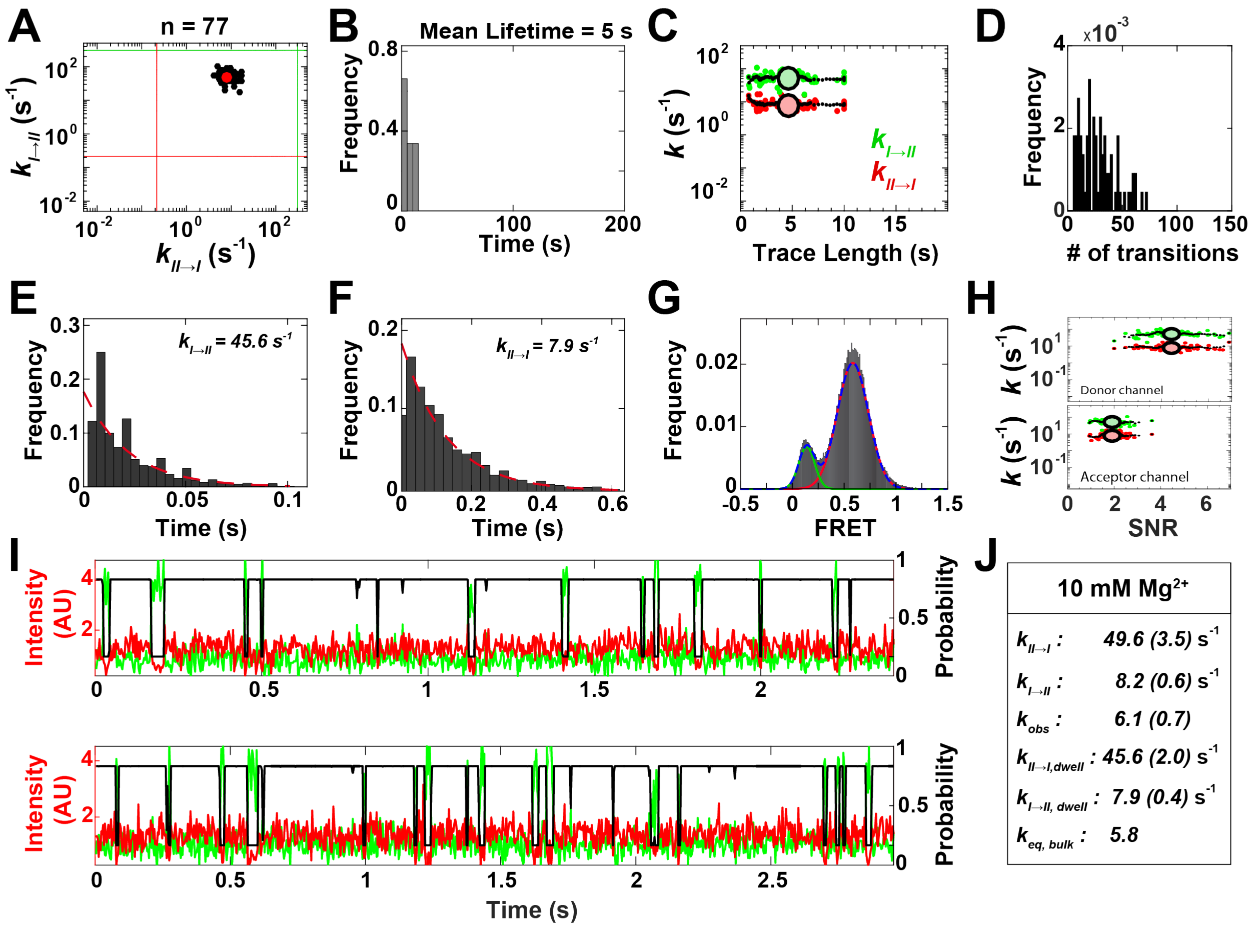

Supplementary Figure S2: SmFRET data analysis and summary statistics. Data shown are from FRET traces of 77 molecules total recorded at high salt (10 mM Mg^{2+}). (**A**) Scatter plot of fitted values of the $k_{I\rightarrow II}$ versus $k_{II\rightarrow I}$ rate constants of individual molecules. Green lines indicate the frame rate of data collection (upper limit) and red lines indicate the mean lifetime of the molecules (lower limit). The red dot is the population median. (**B**) Histogram of lifetimes of the smFRET traces. (**C**) Scatter plots of rate constants versus the length of the smFRET traces. The mean value for each rate constant is represented by a larger circle. Black dots are averages of five values and are shown to provide a visual guide. (**D**) Distribution of the number of transitions per trace. (**E**) and (**F**) Distribution of dwell times in the low (**E**) and high (**F**) FRET states. Exponential fits are shown (red lines) along with the fitted single-exponential rate constants. (**G**) Cumulative FRET distributions. Distribution was fitted to a sum of two Gaussians (blue dashed line). The observed equilibrium constant from cumulative data was determined from the ratio of area under the high (red, FRET = 0.58) and low (green, FRET = 0.14) FRET components of the distribution. (**H**) Rate constants as functions of the signal-to-noise ratio (SNR) in the donor (top) and acceptor (bottom) channels. (**I**) Randomly-selected smFRET traces. The intensities of the donor dye (green) and the acceptor dye (red) are shown. The black line denotes the probability of occupying the high FRET state as determined by a two-state HMM model. (**J**) Summary table of single-molecule and cumulative kinetic and thermodynamic parameters.

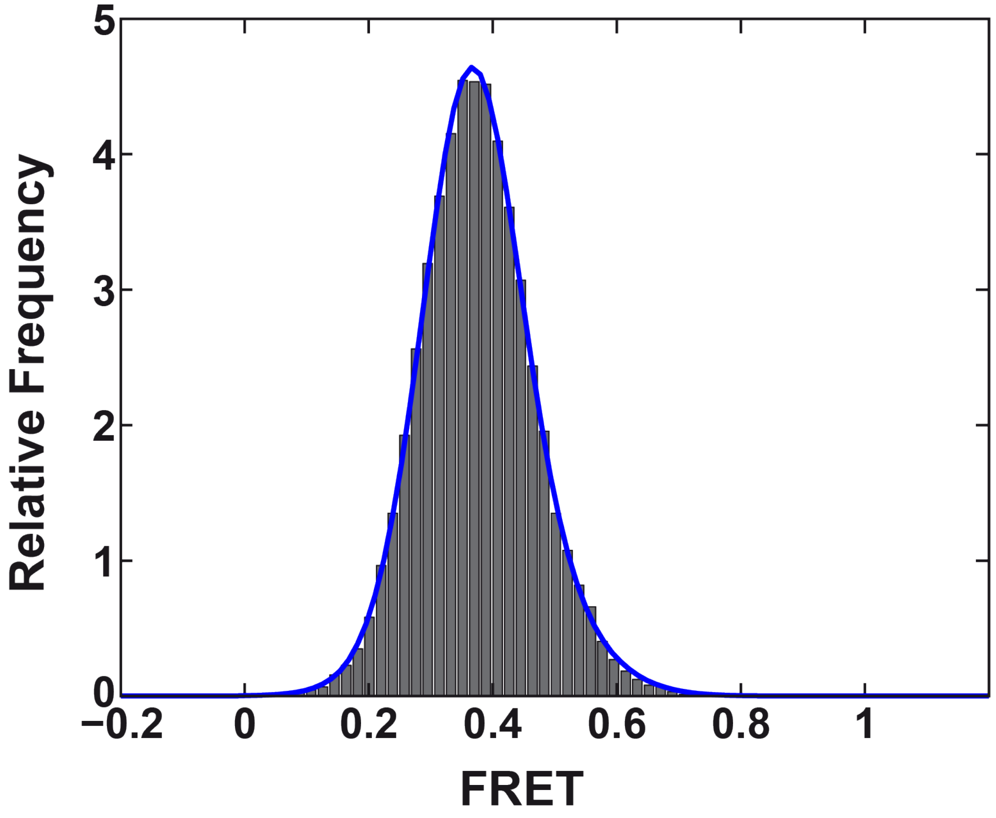

Supplementary Figure S3: FRET distribution at intermediate salt (150 μM Mg^{2+}). The blue line is a Gaussian fit to the distribution.

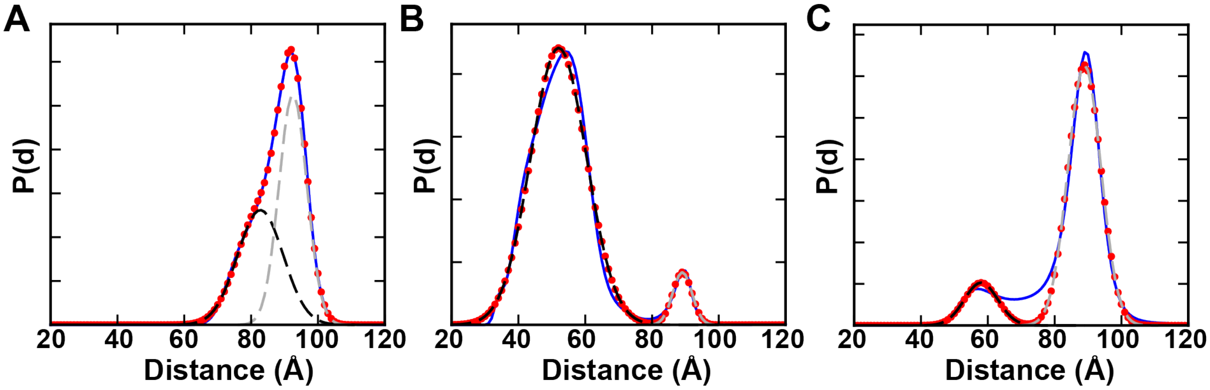

Supplementary Figure S4: XSI distance distributions at 10 mM $MgCl_2$ and 30 mM Tris, pH 7.4. Experimentally determined distance distribution and individual contributions from the two different conformations for label pairs (**A**) BR, (**B**) BH and (**C**) HR. Two Gaussians (dashed black and gray lines) were fitted into the experimental measured distance distributions (blue line) such that the sum of both Gaussians (red circles) minimizes the difference to the experimental distribution. The number of points was reduced for clarity.

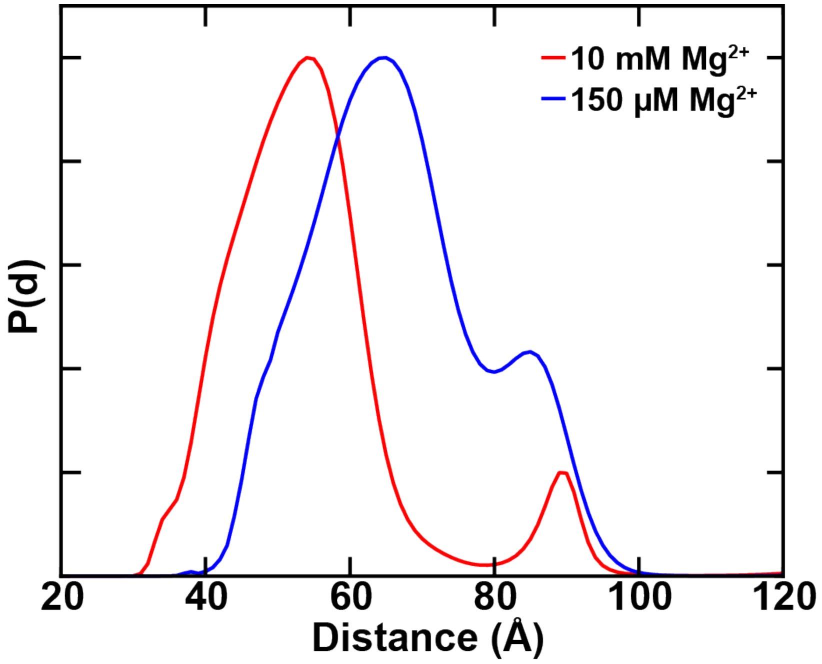

Supplementary Figure S5: XSI distance distributions for the label pair BH recorded at high Mg^{2+} (red, 10 mM $MgCl_2$ and 30 mM Tris-HCl, pH 7.4) and intermediate Mg^{2+} (blue, 150 μM $MgCl_2$ and 30 mM Tris-HCl, pH 7.4).

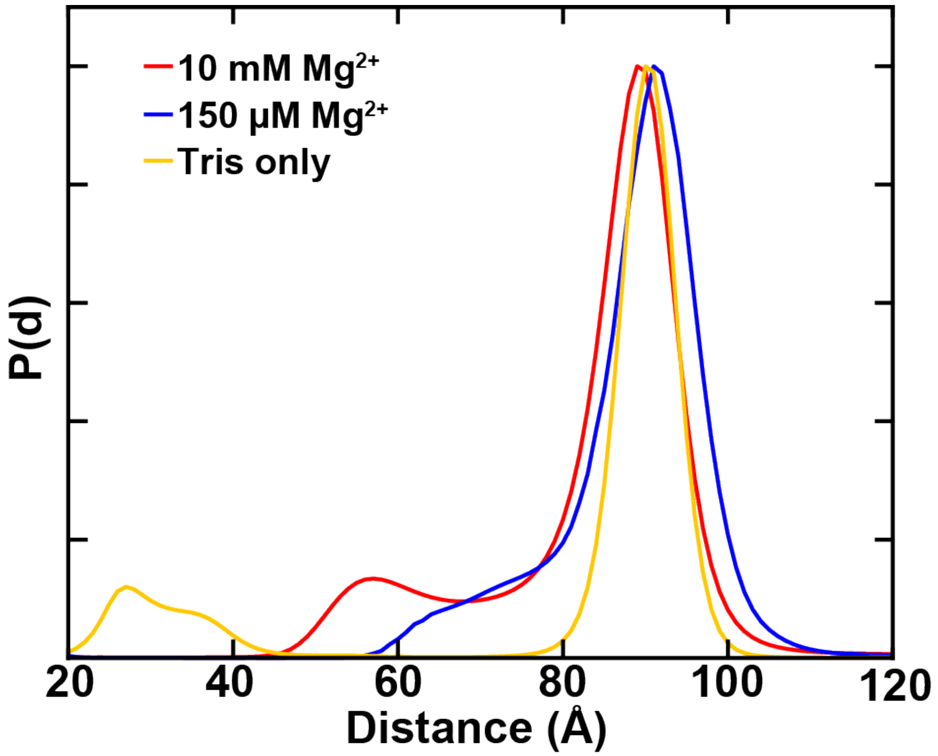

Supplementary Figure S6: XSI distance distributions for the label pair HR recorded at high Mg^{2+} (red, 10 mM $MgCl_2$ and 30 mM Tris-HCl, pH 7.4), intermediate Mg^{2+} (blue, 150 µM $MgCl_2$ and 30 mM Tris-HCl, pH 7.4) and low salt (yellow, 30 mM Tris-HCl, pH 7.4).

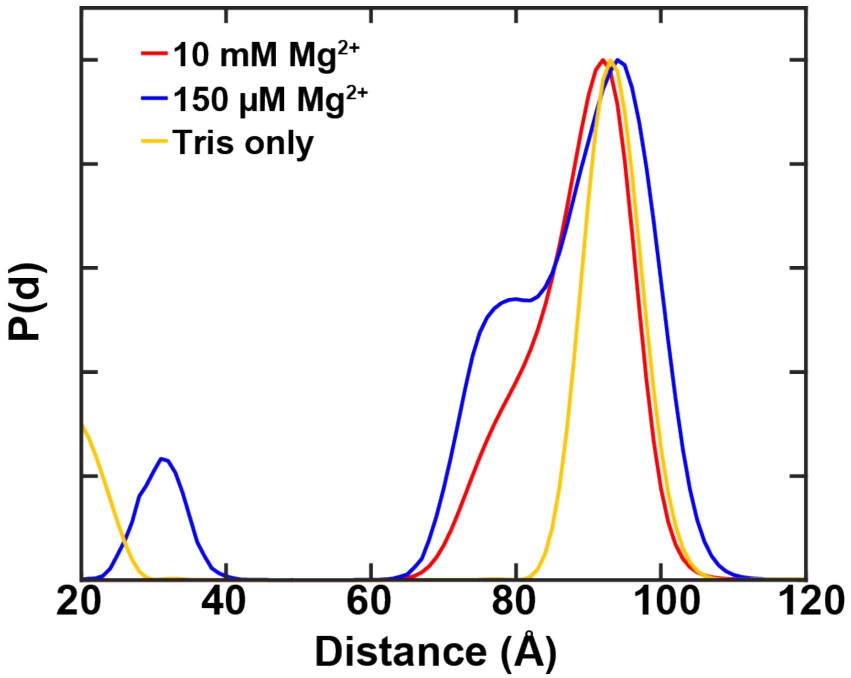

Supplementary Figure S7: XSI distance distributions for the label pair BR recorded at high Mg^{2+} (red, 10 mM $MgCl_2$ and 30 mM Tris-HCl, pH 7.4), intermediate Mg^{2+} (blue, 150 μM $MgCl_2$ and 30 mM Tris-HCl, pH 7.4) and low salt (yellow, 30 mM Tris-HCl, pH 7.4).

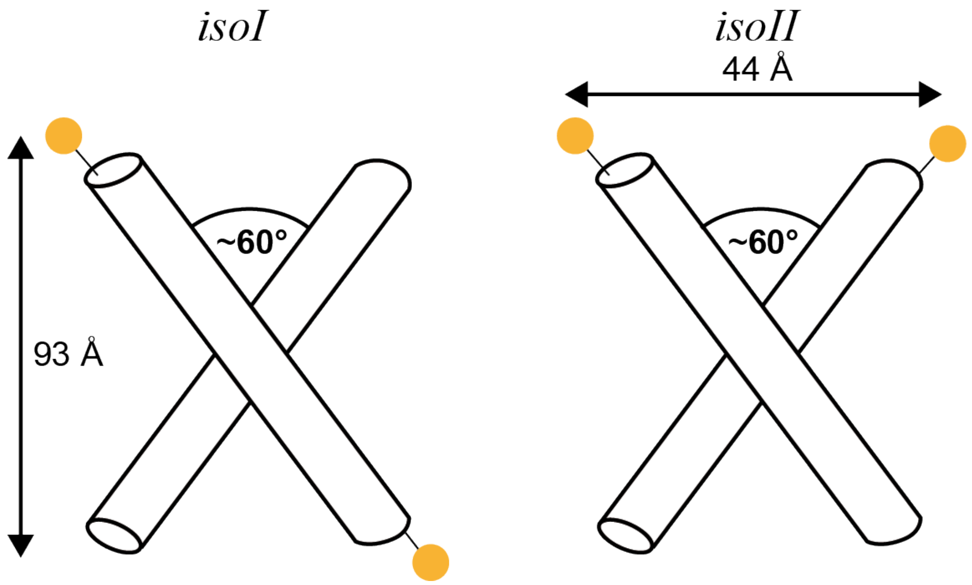

Supplementary Figure S8: Schematic of the proposed X-shape with the BH label pair in *isoI* and *isoII*. The arm length is 11 bp each in our design. The distance between the gold labels in *isoI* were obtained, assuming B-form DNA with a helical rise of 3.3 Å and a 11 Å axial label offset. The distances between the two gold probes in *isoII* were calculated using an isosceles triangle model with the literature value ~60° (inter-duplex angle) and the same parameters as for *isoI*. Based on this model, a shorter label-label distance is expected for *isoII* compared to *isoI*.

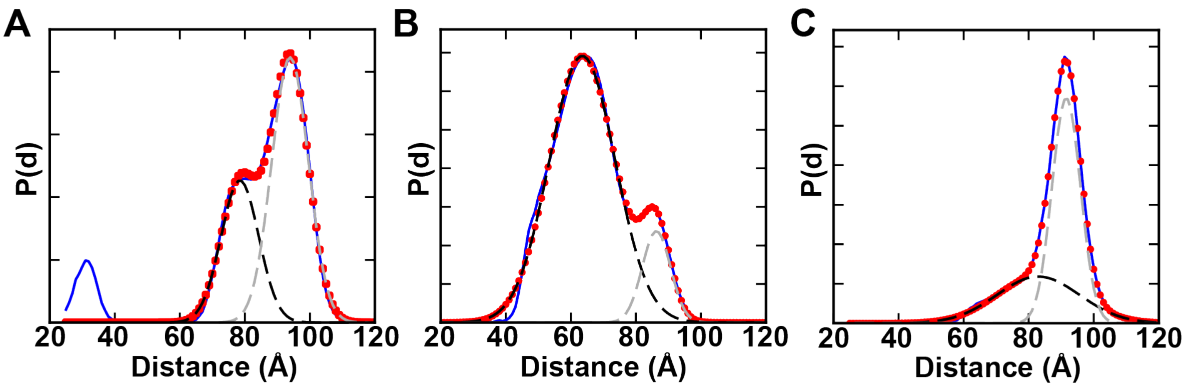

Supplementary Figure S9: XSI distance distributions at 150 µM $MgCl_2$ and 30 mM Tris, pH 7.4. Experimentally determined distance distribution and individual contributions from the two different conformations for label pairs (**A**) BR, (**B**) BH and (**C**) HR. Two Gaussians (dashed black and gray lines) were fitted into the experimental measured distance distributions (blue line) such that the sum of both Gaussians (red circles) minimizes the difference to the experimental distribution. The number of points was reduced for clarity.

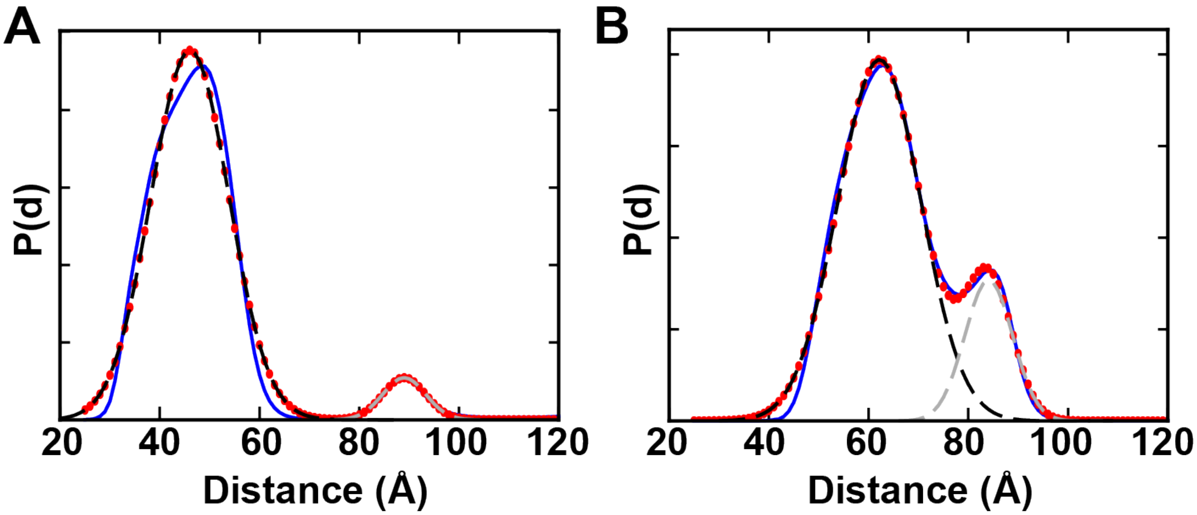

Supplementary Figure S10: XSI distance distributions for the label pair BH recorded at (**A**) 1 M NaCl and 30 mM Tris, pH 7.4 as well as (**B**) 150 mM NaCl and 30 mM Tris, pH 7.4. Two Gaussians (dashed black and gray lines) were fitted to the experimentally measured distance distributions (blue line) such that the sum of both Gaussians (red circles) minimizes the difference to the experimental distribution. The number of points was reduced for clarity.

Table S1. Unmodified DNA sequences used to assemble the Holliday junction. The position of the junction is indicated in bold font.

DNA Strand	Sequence
R	5' – CCCTAGCAAG**GG**GCTGCTACGG
X	5' – CCGTAGCAGC**CT**GAGCGGTGGG
B	5' – CCCACCGCTC**AA**CTCAACTGGG
H	5' – CCCAGTTGAG**TC**CTTGCTAGGG

Table S2. Modified DNA sequences used to assemble the Holliday junction.

DNA Strand	Sequence
R–thiol	5' – CCCTAGCAAGGGGCTGCTACGG – thiol
X-thiol	5' – CCGTAGCAGCCTGAGCGGTGGG – thiol
B-thiol	5' – CCCACCGCTCAACTCAACTGGG – thiol
H-thiol	5' – CCCAGTTGAGTCCTTGCTAGGG – thiol
R-Cy3b	5'–CCCTAGCAAGGGGCTGCTACGG – Cy3b
X-Cy5	5'–CCGTAGCAGCCTGAGCGGTGGG – Cy5
B-biotin	5'–CCCACCGCTCAACTCAACTGGG – biotin

Table S3. Gold label peak distances for the different pairs recorded at various salt conditions. The uncertainties in the positions of the distance peaks are ± 0.5%, based on the standard deviation of repeated measurements for the HR and RX label pairs at Tris only and in agreement with uncertainties reported by Fenn *et al.* (Ref. 24 in the main text). We note that the reported errors are the uncertainty on the position of the peaks, not the width of the peaks, which is larger.

Label pair:	Salt concentration:	First Peak [Å]:	Second Peak [Å]:
BH	10 mM Mg^{2+}	54.0 ± 0.3	89.0 ± 0.4
HR	10 mM Mg^{2+}	57.0 ± 0.3	89.0 ± 0.4
BR	10 mM Mg^{2+}	82.0 ± 0.4	92.0 ± 0.5
BH	150 μM Mg^{2+}	61.0 ± 0.3	84.0 ± 0.4
HR	150 μM Mg^{2+}	65.0 ± 0.3	91.0 ± 0.5
BR	150 μM Mg^{2+}	78.0 ± 0.4	94.0 ± 0.5
BH	1 M Na^{+}	46.0 ± 0.2	88.0 ± 0.4
BH	150 mM Na^{+}	63.0 ± 0.3	84.0 ± 0.4
HR	Tris only	90 ± 0.5	-
RX	Tris only	86.5 ± 0.5	-
BR	Tris only	93.0 ± 0.5	-

Table S4. Population of *isoI* and *isoII* obtained from Gaussian fits to XSI data at 10 mM $MgCl_2$.

**Label pair:**	***isoI*** **:**	***isoII:***
BH	6.1%	93.9%
HR	14.3%	85.7%
BR	28.7%	71.3%
Average	16.3%	83.7%
